# Vascular Access Site for Renal Replacement Therapy in Acute Kidney Injury: A *Post hoc* Analysis of the ATN Study

**DOI:** 10.3389/fmed.2017.00040

**Published:** 2017-04-11

**Authors:** Yue-Harn Ng, Kavitha Ganta, Herbert Davis, V. Shane Pankratz, Mark Unruh

**Affiliations:** ^1^Division of Nephrology, Department of Internal Medicine, University of New Mexico, Albuquerque, NM, USA; ^2^New Mexico VA Health Care System, Albuquerque, NM, USA; ^3^Department of Internal Medicine, University of New Mexico, Albuquerque, NM, USA

**Keywords:** acute kidney injury, complications, dialysis catheter, dialysis dose, mortality

## Abstract

**Background:**

Acute kidney injury requiring renal replacement therapy (RRT) in the intensive care unit portends a poor prognosis. The decisions regarding dialysis catheter placement is based mainly on physician discretion with little evidence to support the choice of dialysis catheter location.

**Methods:**

The Veterans Affairs/National Institutes of Health Acute Renal Failure Trial Network Study was a multicenter, prospective, randomized trial of intensive vs. less intensive RRT in critically ill patients with AKI. We assessed the association of dialysis catheter location with dialysis catheter-related outcomes including catheter-related complications, mortality, dialysis dependence, and dialysis dose delivered.

**Results:**

Of the 1,124 patients enrolled in the ATN study, catheter data were available in 1,016 (90.39%) patients. A total of 91 (8.96%) subclavian, 387 (38.09%) internal jugular, and 538 (52.95%) femoral dialysis catheters were inserted. The femoral group was younger (58.39 ± 16.27), had greater bleeding tendency [lower platelet count (96.00 ± 109.35) with higher INR (2.01 ± 2.19)], and had a higher baseline sequential organ failure assessment score on admission (14.59 ± 3.61) compared to the other two groups. Dialysis catheter-related complications were low in this study with no significant difference in the rates of complications among all catheter locations. Mortality and dialysis dependence was lowest in the subclavian group, while the dose of dialysis delivered (Kt/V) remained lowest in the femoral group, after propensity score and center adjustments.

**Conclusion:**

Patient characteristics influence the choice of dialysis catheter location with a tendency to place femoral catheters in younger, sicker, and more coagulopathic patients. There were no statistically significant differences in complication rates among the three catheter locations, although femoral catheters may be associated with a lower delivered dose of dialysis during intermittent hemodialysis.

**Clinical Trial Registration:**

www.ClinicalTrials.gov, identifier NCT00076219.

## Introduction

Establishing temporary vascular access is essential for patients with acute kidney injury requiring renal replacement therapy (RRT) (1). The placement of a central venous dialysis catheter, however, may negatively affect the morbidity and mortality of patients with AKI, especially in the intensive care unit (ICU) setting. Mechanical complications such as inadvertent arterial puncture, which can result in excessive bleeding or thrombotic complications, pneumothorax, hemothorax, air embolism as well as cardiac arrhythmia are infrequent but may be potentially fatal especially in the critically ill patients (2). Furthermore, the presence of a catheter increases the risk of catheter-related bloodstream infection and catheter malfunction (3). The frequencies of these complications depend on catheter factors including catheter location (internal jugular vs. femoral vs. subclavian), duration of catheter placement; patient factors including body habitus, comorbidities, patient coagulopathies, and the severity of patient illness as well as operator experience. Placement of subclavian dialysis catheters has been strongly discouraged due to the high incidence of subclavian vein stenosis post catheter insertion (4, 5). Femoral catheters, on the other hand, have been associated with deep venous thrombosis (6, 7).

Several guidelines exist with recommendations for dialysis catheter placements in the setting of AKI including location, duration, and types of catheter placement although none are supported by strong evidence and generally suggest using internal jugular site for acute hemodialysis mainly to reduce infection risk and for patient comfort (8–10). The most recent CDC guideline recommends that femoral central line placement should be avoided (10). The 2006 KDOQI guidelines suggest internal jugular catheters should not be used for more than a week, while femoral catheters should be left in place for no more than 5 days and only in bed-bound patients due to the high risk of dislodgement (9). In the KDIGO guideline for AKI published in 2012, it was suggested that the first choice for dialysis catheter placement should be the right internal jugular, followed by femoral, and then left internal jugular (8). Historical data suggest that femoral catheters have a higher risk of infection due to the proximity to the groin (2, 11). This assumption, however, has been disproven in the Cathedia study, which was a large randomized, controlled trial comparing the risk of nosocomial events in femoral vs. jugular venous catheterization in AKI patients (12). The study concluded that the risk of nosocomial infection was comparable between jugular and femoral catheters except for patients with high BMI, whereby jugular placement was associated with a lower risk of infection. The Cathedia study remains the largest and only randomized controlled trial to date looking at the effect of dialysis catheter location on infection; however, the study focused mainly on catheter-related infections and did not specifically address the impact of location of catheter on other outcomes of the catheter such as dialysis adequacy, complication rates such as pneumothorax, hemothorax, and bleeding as well as mortality and dialysis dependence.

To better understand the relationship of catheter site to these key outcomes, we used the Veterans Affair/National Institute of Health Acute Renal Failure Trial Network study, which was a multicenter, prospective, randomized parallel-group trial assessing the effect of intensity of hemodialysis on mortality in critically ill patients with AKI (13). We took advantage of the ATN study to test whether complication rates, dialysis dose delivered, mortality, and dialysis dependence were related to three different catheter locations in critically ill patients requiring RRT.

## Materials and Methods

### Study Participants

Patients enrolled in the ATN study were critically ill adults (18 years or older) who had AKI clinically consistent with acute tubular necrosis and failure of one or more non-renal organ [defined as a non-renal sequential organ failure assessment (SOFA) score of ≥2]. Inclusion and exclusion criteria are available in the original VA/NIH Acute Renal Failure Trial manuscript (13). The Institutional Review Board (IRB) of the University of New Mexico approved this secondary analysis of the ATN Trial. All study participants or their health-care surrogates provided informed consent to participate in ATN, and the ethics committees/IRBs of participating centers had reviewed and approved the consent form during protocol review. These documents and the ATN protocols can be downloaded from the NIDDK repository. Individual ATN participants were not consented for this secondary analysis, because the data as distributed by the NIDDK have been de-identified. Furthermore the data use agreement between the investigators of this paper and NIDDK prohibits us from making any contact to identify individuals, families, or communities. The IRB of the University of New Mexico waived the requirement for an informed consent for this secondary analysis after reviewing the original consent form that ATN participants signed upon their enrollment, the data use agreement between the investigators and NIDDK, and the associated research protocol submitted to the NIDDK.

### Catheter Placement

In this study, the site of dialysis catheter insertion and indication for removal of catheter was based on physician discretion. Data captured during catheter insertion process included date and time of catheter insertion, number of catheters inserted per patient, temporary or tunneled catheter placement, location of catheter, and insertion complications including early and late complications (late being defined as complications occurring at >24 h from catheter insertion to 3 days after catheter removal). Date and time as well as reason for catheter removal were also captured. Only first catheter placements were included in the primary analysis. Recurrent catheter insertion and complications were not assessed in this study.

### Complications

Complications collected related to catheter placement included infection, cardiac arrhythmia, pneumothorax, hemothorax, arterial puncture, local venous thrombosis, and air embolism.

### Hemodialysis and Patient Outcomes

We looked at the associations among the three different catheter locations on catheter function, duration of catheter placement, and dialysis dose delivered. We attempted to address the issue of catheter malfunction by looking at a combination of factors, acknowledging that no one factor can be used to define cateter malfunction, including the blood flow rate achieved during dialysis, the number of filters changed in a 24-h period, and the incidence of clotting events that required filter change. In addition, we assessed the association between the choice of catheter location on patient outcomes including 60-day mortality and dialysis dependence.

### Statistics

Primary comparisons of baseline patient characteristics among the three groups were defined by the location of the initial catheter placement and were performed using analyses of variance for continuous variables and chi-square tests for categorical variables. To account for renal recovery as a potential competing risk factor for mortality, we looked at the combined outcome of real recovery and mortality. Comparisons of the combined renal recovery and 60-day mortality outcomes among the catheter placement groups were made using multinomial regression models. Analyses for Kt/V were accomplished using repeated measures analysis of variance. Because catheter placement was not randomized, and was influenced by the clinicians’ perceptions of their patients, we repeated the analyses while attempting to correct for differences among groups *via* the application of propensity weights. Propensity weights were estimated by the twang (version 1.4-9.3) (14) package in R (version 3.2.3) using all baseline covariates as shown in Table 1. As there were individuals with missing data, we performed a multiple imputation approach for missing values of the patient characteristics using the “chained equations” approach (15). This method fits a regression model for each variable with missing data, conditional on other variables, and makes a prediction for each missing value from the fitted regression model. Ten data sets with complete data were generated, and propensity weights appropriate for estimating the average treatment effect were obtained from each data set. The geometric mean of the estimated propensity weights was obtained for each participant across the 10 imputed data sets. Analyses were repeated to compare the outcomes of interest among the groups with different initial catheter placement locations while weighting the individuals by their estimated propensity scores. Subsequent analyses adjusted for the different study sites were also performed using generalized estimating equations approaches. All analyses with the exception of the propensity weights were computed using SAS version 9.4 (Cary, NC, USA) and R (version 3.2.3).

**Table 1:** **Baseline characteristics**.

	Catheter location			*P* values
	Subclavian (*n* = 92)	Internal jugular (*n* = 387)	Femoral (*n* = 538)	
**Baseline characteristic**				
Age, year	59.82 ± 14.24	61.62 ± 14.22	58.39 ± 16.27	<0.01
Male	65 (70.65)	282 (72.68)	368 (68.40)	0.37
BMI	26.86 ± 6.13	28.96 ± 6.34	27.66 ± 5.76	<0.01
**Comorbidities**				
Liver disease	9 (9.78)	45 (11.60)	64 (11.90)	0.52
Diabetes	35 (38.04)	118 (30.41)	146 (27.14)	0.08
Hypertension	5 (5.43)	13 (3.35)	16 (2.99)	0.32
PVD	24 (26.09)	85 (21.91)	62 (11.52)	<0.01
CVD	1 (1.09)	2 (0.52)	3 (0.56)	0.72
CHF	23 (25.00)	104 (26.80)	118 (21.93)	0.23
CVA	7 (7.61)	38 (9.79)	49 (9.11)	0.8
**Laboratory values**				
WBC (× 10^2^ cells/mm^3^)	14.85 ± 9.53	12.50 ± 10.01	13.40 ± 11.89	0.14
Platelets (× 10^3^ cells/mm^3^)	101.00 ± 146.77	114.50 ± 111.49	96.00 ± 109.35	<0.01
INR	1.61 ± 1.04	1.64 ± 0.78	2.01 ± 2.19	<0.01
BUN (mg/dL)	66.57 ± 32.84	70.41 ± 33.81	61.46 ± 33.32	<0.01
Creatinine (mg/dL)	3.92 ± 1.53	4.26 ± 1.77	3.98 ± 2.51	0.02
Albumin (g/dL)	2.34 ± 0.69	2.48 ± 0.87	2.36 ± 0.73	0.08
**Clinical parameters**				
Sepsis	54 (64.29)	179 (48.38)	289 (57.23)	<0.01
MAP (mmHg)	74.17 ± 14.26	73.89 ± 13.62	73.86 ± 15.39	0.98
Edema	23 (71.88)	88 (67.18)	63 (57.27)	0.3
Intubation	75 (81.52)	293 (75.52)	455 (84.57)	<0.01
SOFA	13.26 ± 3.75	13.07 ± 3.79	14.59 ± 3.61	<0.01

*MAP, mean arterial pressure; PVD, peripheral vascular disease; CVD, cardiovascular disease; CHF, congestive heart failure; CVA, cerebrovascular accidents; SOFA, sequential organ failure assessment*.

## Results

A total of 1,124 patients were enrolled in the ATN study between November 2003 and July 2007. Data on dialysis catheter placement was available in 1,016 (90.39%) of the study patients. A total of 91 (8.96%) subclavian catheters, 387 (38.09%) internal jugular catheters, and 538 (52.95%) femoral catheters were placed.

### Baseline Characteristics

Table 1 demonstrates the baseline characteristics of the patients by catheter site. The femoral group was younger, more coagulopathic with a higher INR, and sicker with a higher baseline SOFA score compared to the other two groups. There were comparable number of patients with documented edema and congestive heart failure. The internal jugular group had the highest BMI among the three groups. Comorbidities including diabetes, hypertension, cardiovascular disease, cerebrovascular accidents, and liver disease were comparable among the three groups with the exception of peripheral vascular disease (PVD) with the femoral group having less PVD compared to the other groups.

There were more patients who were intubated in the femoral group compared to the subclavian and internal jugular group. The use of inotropic agents was, however, comparable among the three groups. The femoral group was started on RRT at a lower BUN compared to the other two groups.

### Outcomes

#### Catheter Complications

The reported rates of catheter-related complications were uniformly low in this study. There were no reported cases of hemothorax and air embolism. Cardiac arrhythmia was most commonly reported in the internal jugular group although this difference did not reach statistical significance. Other complications including inadvertent arterial puncture, bleeding, excessive bleeding, and venous thrombosis were comparable among the three groups (Table 2).

**Table 2:** **Catheter-related complications**.

	Subclavian	Internal jugular	Femoral	*P* values
Arterial puncture	0 (0)	1 (0.26)	3 (0.56)	0.63
Bleeding	0 (0)	9 (0.19)	5 (0.09)	0.15
Excessive bleed	1 (1.09)	3 (0.77)	4 (0.74)	0.94
Late excessive bleed	2 (2.17)	3 (0.77)	2 (0.37)	0.15
Cardiac arrhythmia	0 (0)	5 (1.41)	2 (0.40)	0.17
Local venous thrombosis	0 (0)	0 (0)	1 (0.19)	0.64
Late local thrombosis	0 (0)	0 (0)	3 (0.56)	0.26

In this study, 19.57% of all enrolled patients had blood cultures obtained, and 26.58% of the catheter tips were sent for culture upon catheter removal. The incidence of bacteremia as well as catheter-related infections was comparable among all catheter groups (Table 3). The most commonly cultured organism was *Staphylococcus epidermidis* followed by methicillin-resistant *Staphylococcus aureus* and vancomycin-resistant *Enterococcus*.

**Table 3:** **Catheter-related infections**.

	Subclavian	Internal jugular	Femoral	*P* values
**Unadjusted (incidence per 1,000 catheter days)**				
Bacteremia (95% CI)	6.7 (3.8–11.9)	4.6 (3.2–6.5)	2.6 (1.8–3.7)	0.009
Catheter-related infections (95% CI)	2.2 (0.8–6.0)	2.0 (1.2–3.4)	0.9 (0.5–1.7)	0.1
**Propensity score adjustment (incidence per 1,000 catheter days)**				
Bacteremia (95% CI)	5.8 (3.6–9.3)	4.8 (3.4–6.7)	5.7 (4.0–8.2)	0.72
Catheter-related infections (95% CI)	3.1 (1.6–5.9)	2.2 (1.3–3.6)	2.4 (1.4–4.2)	0.72

#### Catheter Malfunction and Dialysis Dose

We assessed the functionality of the dialysis catheter by assessing a combination of factors including the blood flow achieved during dialysis, the duration of placement for each catheter, the dose of dialysis delivered, and the frequency at which the filters clotted or were changed in each catheter group. The femoral group had the shortest duration of catheter placement and the lowest Kt/V compared to the other groups. The subclavian group reported a higher rate of clotting compared to the femoral and internal jugular group both during continuous renal replacement therapy (CRRT) and intermittent hemodialysis (IHD) although the number of filters changed within a 24-h period was not clinically different. Interestingly, the blood flow rate achieved was the lowest in the subclavian group during CRRT, whereas it was the highest during IHD. The dose of dialysis delivered during IHD was lowest in the femoral group, and this difference persisted even after propensity score adjustments (Table 4).

**Table 4:** **Dialysis dose and catheter malfunction**.

	Subclavian	Internal jugular	Femoral	*P* values
**Continuous renal replacement therapy**				
Blood flow (mL/min)	135.00 ± 31.72	150.00 ± 32.07	150.00 ± 34.01	<0.01
Dialyzate flow (mL/h)	1,250.00 ± 499.58	1,050.00 ± 466.33	1,150.00 ± 482.66	0.04
Duration (h)	19.00 ± 6.92	20.00 ± 7.09	20.00 ± 6.91	<0.01
Effluent volume (L/day)	34.00 ± 22.32	39.00 ± 23.38	39.30 ± 21.44	<0.01
CVVHDF clot^a^	209 (51.48)	730 (38.34)	994 (40.59)	<0.01
Anticoagulation	538 (51.83)	2,333 (50.17)	3,090 (54.17)	<0.01
**Intermittent hemodialysis**				
Blood flow (mL/min)	400.00 ± 70.08	350.00 ± 62.82	350.00 ± 75.74	<0.01
DFR (mL/min)	800.00 ± 127.36	800.00 ± 153.48	800.00 ± 183.08	<0.01
Duration (h)	4.00 ± 0.94	4.00 ± 1.66	4.00 ± 2.64	<0.01
Hemo clot^b^	47 (14.83)	169 (9.30)	263 (13.75)	<0.01
Anticoagulation	463 (44.61)	2,229 (47.94)	2,504 (43.90)	<0.01
Kt/V	1.27 ± 0.38	1.32 ± 0.37	1.24 ± 0.34	<0.01
Duration of catheter (days)	7.90 ± 7.62	8.30 ± 5.75	6.42 ± 4.74	<0.01
**Propensity score adjustment**				
Kt/V	1.21 ± 0.04	1.29 ± 0.04	1.20 ± 0.04	<0.01

*Blood flow, dialyzate flow, duration of dialysis, and effluent volume are expressed as median ± SD*.

*^a^CVVHDF clot: CVVHDF clotting requiring hemodiafilter replacement*.

*^b^Hemo clot: clotting requiring hemodialyzer replacement*.

#### Patient Outcomes

**Figure 1:**
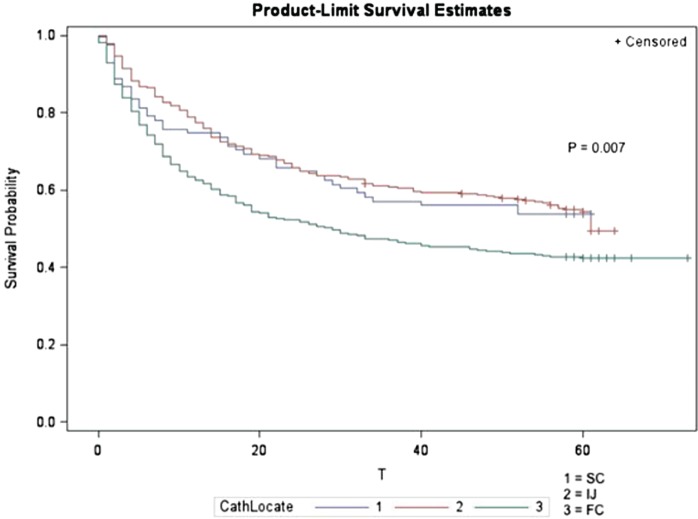
**Unadjusted survival curve by catheter location**.

The 60-day mortality rate in this study population was 45.64%. As noted in Figure 1, there was a significant difference in mortality among the three catheter locations. To address the question of whether this difference in mortality seen was due to the unequal distribution of the patient population and to account for renal recovery as a potential competing risk factor, we analyzed the effect of catheter location on mortality and dialysis dependence using a multinomial regression model.

**Figure 2:**
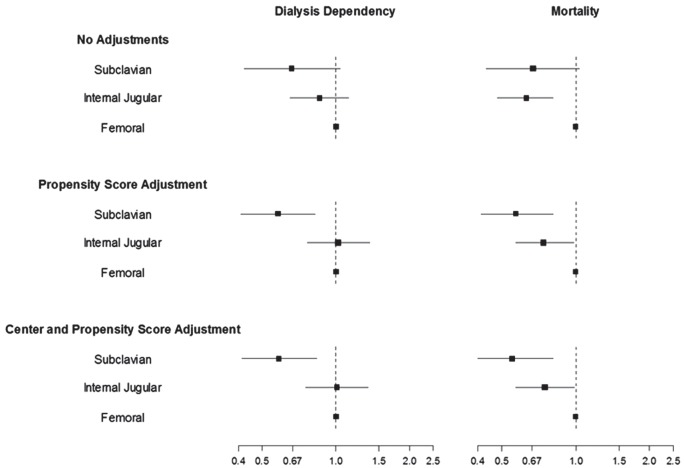
**Catheter location and outcomes**.

Patient mortality remained lower in the subclavian group (OR 0.55; 95% CI 0.4–0.81) and the internal jugular group (OR 0.75; 95% CI 0.57–0.98) when compared to the femoral group, while dialysis dependence was noted to be lower in the subclavian group compared to the femoral group (OR 0.58; 95% CI 0.41–0.83) with no significant difference between the internal jugular group and the femoral group (OR 1.01; 95% CI 0.75–1.35) after propensity score and center adjustment (Figure 2).

## Discussion

### Key Findings

Our study demonstrated that complications related to catheter placement were low and fairly comparable across the three catheter sites, while lower delivered dose of dialysis during IHD was seen in the femoral group compared to the SC and IJ group. In this study, we also showed that patient characteristics influence the choice of dialysis catheter location with a tendency to place femoral catheters in younger, sicker, and more coagulopathic patients, which may have influenced the rates of mortality and renal recovery.

Sixty-day mortality and dialysis dependence was noted to be lowest in the subclavian group compared to the femoral and internal jugular groups even after propensity score and center adjustment. The distribution of propensity scores in the subclavian group was considerably different from the distributions of the propensity scores in the internal jugular and femoral groups. This suggests that patients who received a subclavian catheter were markedly different at baseline compared to the other two catheter groups, and the current findings on lower mortality and dialysis dependence in this group may reflect the difference in the patient population who received a subclavian catheter. We have attempted to address this shortcoming by using propensity weights although there may be factors that we could not adequately account for. The choice of dialysis catheter location will likely continue to depend on physician experience and patient characteristics, for example, patients with high BMI receiving internal jugular catheters. Femoral catheters often are, and likely will remain, the site of choice in the cases of emergencies (1, 16).

### Relationship to Previous Studies

In this study, however, we did demonstrate that the difference in catheter location impacts catheter function and dialysis dose delivered. We acknowledge that there is no single parameter that determines catheter functionality, and certain parameters may be influenced by physician decisions, hence we looked at a combination of factors including catheter blood flow rates, duration of catheter usage, dialyzer clotting resulting in filter change as well as dialysis dose delivered (17, 18). In comparison to the Cathedia study, we noted a higher incidence of catheter malfunction in the femoral group compared to the other groups as shown by the shorter catheter duration and lower delivered Kt/V (19). One potential explanation for this difference may be the exclusion of patients who could not be randomized due to medical reasons in the Cathedia study (e.g., coagulopathic patients in whom jugular catheter insertion may result in a higher risk for bleeding) hence eliminating patients who could be at higher risk for catheter malfunction in that trial.

Two other randomized studies, performed in critically ill patients in the ICU, both found a higher rate of venous thrombosis in the femoral group (1, 20). However, these studies involved central venous catheter placement and not dialysis catheter placement, hence the issue of catheter malfunction and dialysis dose was not assessed. Studies looking specifically at dialysis catheter placements have also found that femoral catheters to be associated with high risk for venous thrombosis (11, 21, 22), more recirculation (23, 24), and catheter malfunction. However, like our study, these studies were not randomized controlled trials. To our knowledge, we are the first to document that femoral catheters may be associated with a lower delivered dose of dialysis during IHD (Kt/V). Dialysis dose in patients with acute kidney injury requiring CRRT has been established based on the VA/NIH ATN study (13) and the RENAL study (25). However, the prescribed Kt/V for AKI patients on IHD has not been clearly determined. There is evidence to suggest that higher dose of dialysis in AKI patient on IHD may enhance survival (26–29), although this has not been proven in randomized controlled trials. The implication of our finding is intriguing and may suggest that when using femoral catheters for dialysis, a higher dose of dialysis may need to be prescribed to overcome the lower dose of dialysis delivered during IHD with this catheter location.

### Strengths and Limitations of the Study

The VA/NIH ATN study was a large, multicentered study with patients recruited from multiple sites throughout the US. The heterogeneity of the population allows for greater applicability of this study to the general ICU population. The prospective collection of a significant amount of outcomes data and the clear definition of each outcome collected from this study also allowed us to study the various complications and outcomes associated with the different dialysis catheter locations and to adjust for numerous factors that could potentially affect the outcomes.

The interpretation of these findings should be performed in light of several important limitations. First, this is a secondary data analysis of a clinical trial, and we note the lack of randomization of our study for catheter placement. We addressed this shortcoming by using statistical approaches to attenuate potential biases. As catheter placement was not the main intervention of interest in this study, there was no standard protocol for who would place the catheter and how the catheters were placed, whether ultrasound guidance was used during insertion, and certain catheter data that could have influenced dialysis dose delivered including lumen size and catheter length were not collected. This could potentially result in different rates of complication due to operator experience and also affected the outcome measures. However, in this study the complication rates were low and comparable among the groups. Another potential weakness in this study is the reliance on center reporting for complications, as there is a potential for underreporting of outcomes. However, given the data collected was part of the prospective trial for a different study intervention, the likelihood of this bias was low.

## Conclusion

In conclusion, the femoral catheter site is a valuable location especially in the setting of emergency catheter placement or in patients with coagulopathy; hence, it is unlikely that we will abandon it as a catheter placement site. However, this study highlights caution in the use of femoral site for dialysis catheter placement as it may result in a lower delivered dose of dialysis especially during IHD. As a result, judicious use of anticoagulation and/or augmentation in the dose of dialysis prescribed may be necessary in order to deliver comparable efficacy of dialysis with other catheter locations.

## Ethics Statement

This study was approved by the institutional review board at the University of New Mexico.

## Author Contributions

Y-HN, KG, and MU have been involved in the design, plan of analysis, interpretation of the data, and the writing of the manuscript, while SP and HD were involved in the statistical analysis of the study. All authors have read and approved this manuscript for submission.

## Conflict of Interest Statement

The authors declare that the research was conducted in the absence of any commercial or financial relationships that could be construed as a potential conflict of interest. The reviewer, AK, and handling editor declared their shared affiliation, and the handling editor states that the process nevertheless met the standards of a fair and objective review.
